# Author Correction: Deciphering sex-specific miRNAs as heat-recorders in zebrafish

**DOI:** 10.1038/s41598-024-55491-x

**Published:** 2024-02-28

**Authors:** Tosca A. van Gelderen, Jérôme Montfort, José Antonio Álvarez-Dios, Violette Thermes, Francesc Piferrer, Julien Bobe, Laia Ribas

**Affiliations:** 1 Institut de Ciències del Mar, Consejo Superior de Investigaciones Científicas (ICM-CSIC), 08003 Barcelona, Spain; 2 PhD Program in Genetics, Autonomous University of Barcelona, 08193 Bellaterra, Spain; 3 Laboratoire de Physiologie et Génomique des Poissons, INRAE, Rennes, France; 4 Departamento de Matemática Aplicada, Facultad de Matemáticas, Universidad de Santiago de Compostela, 15781 Santiago de Compostela, Spain

Correction to: *Scientific Reports* 10.1038/s41598-022-21864-3, published online 04 November 2022

The original version of this Article omitted an affiliation for Tosca A. van Gelderen. Their correct affiliations are listed below.

Institut de Ciències del Mar, Consejo Superior de Investigaciones Científicas (ICM-CSIC), 08003, Barcelona, Spain.

PhD Program in Genetics, Autonomous University of Barcelona, 08193, Bellaterra, Spain.

The original Article has been corrected.

