# Supplementary material for: Deciphering sex-specific miRNAs as heat-recorders in zebrafish
Source: Sci Rep. 2022 Nov 4;12:18722. doi: 10.1038/s41598-022-21864-3 (PMC9636255; doi:10.1038/s41598-022-21864-3)
Supplement: Supplementary file 1 — Supplementary Information 1. [file 41598_2022_21864_MOESM1_ESM.pdf]

Dataset 1. Quality of RNA samples assessed by BioAnalyzer (2100 Bioanalyzer, Agilent Technologies) showing RNA Integrative Number (RIN) values for all the testicular samples:A-D control and E-H treated and ovarian samples: A-D control and E-H treated.

A

TCT1

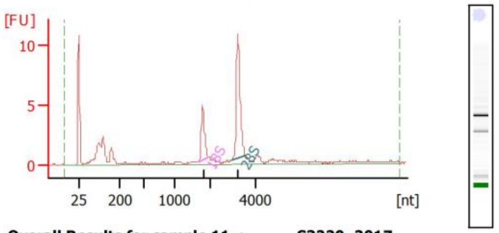

Overall Results for sample 11 : S3220_2017

RNA Area: 57,1
RNA Concentration: 36 ng/µl
rRNA Ratio [28s / 18s]: 0,0
RNA Integrity Number (RIN): 9.6 (B.02.08)
Result Flagging Color:
Result Flagging Label: RIN: 9.60

B

TCT2

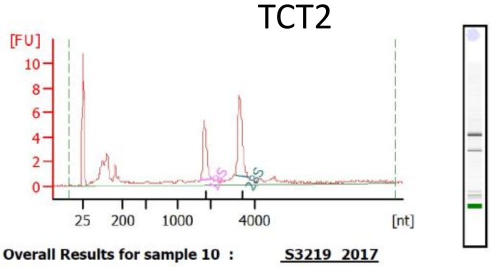

Overall Results for sample 10 : S3219_2017

RNA Area: 62,6
RNA Concentration: 39 ng/µl
rRNA Ratio [28s / 18s]: 0,0
RNA Integrity Number (RIN): 8.7 (B.02.08)
Result Flagging Color:
Result Flagging Label: RIN: 8.70

C

TCT3

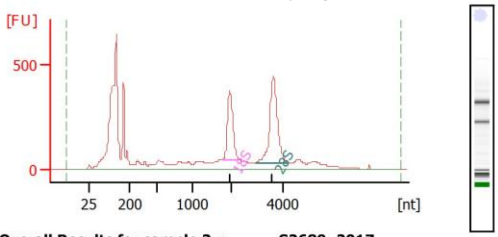

Overall Results for sample 2 : S2689_2017

RNA Area: 7.603,4
RNA Concentration: 16.658 pg/µl
rRNA Ratio [28s / 18s]: 0,0
RNA Integrity Number (RIN): 8.1 (B.02.08, Anomaly Threshold(s) manually adapted)
Result Flagging Color:
Result Flagging Label: RIN: 8.10

D

TCT4

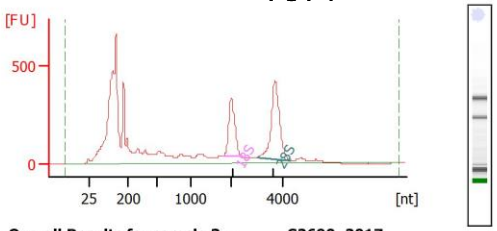

Overall Results for sample 3 : S2690_2017

RNA Area: 8.652,8
RNA Concentration: 18.957 pg/µl
rRNA Ratio [28s / 18s]: 0,0
RNA Integrity Number (RIN): 7.6 (B.02.08, Anomaly Threshold(s) manually adapted)
Result Flagging Color:
Result Flagging Label: RIN: 7.60

E

THT9

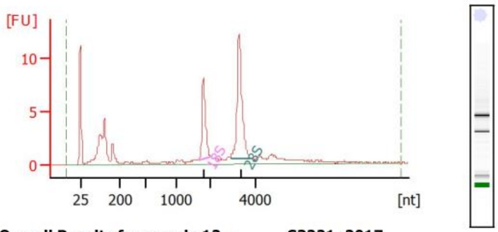

Overall Results for sample 12 : S3221_2017

RNA Area: 79,6
RNA Concentration: 50 ng/µl
rRNA Ratio [28s / 18s]: 0,0
RNA Integrity Number (RIN): 9.5 (B.02.08)
Result Flagging Color:
Result Flagging Label: RIN: 9.50

F

THT10

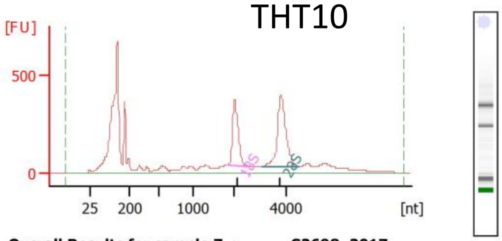

Overall Results for sample 7 : S2698_2017

RNA Area: 7.438,4
RNA Concentration: 16.297 pg/µl
rRNA Ratio [28s / 18s]: 0,0
RNA Integrity Number (RIN): 8.2 (B.02.08, Anomaly Threshold(s) manually adapted)
Result Flagging Color:
Result Flagging Label: RIN: 8.20

G

THT11

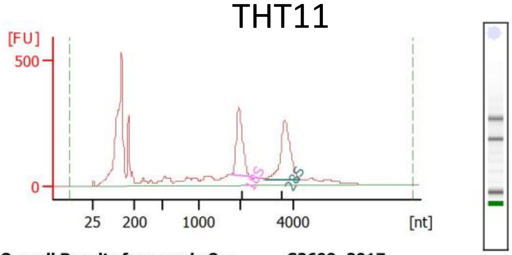

Overall Results for sample 8 : S2699_2017

RNA Area: 5.855,7
RNA Concentration: 12.829 pg/µl
rRNA Ratio [28s / 18s]: 0,0
RNA Integrity Number (RIN): 7.9 (B.02.08)
Result Flagging Color:
Result Flagging Label: RIN: 7.90

H

THT12

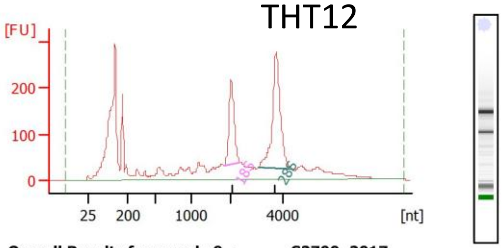

Overall Results for sample 9 : S2700_2017

RNA Area: 4.259,8
RNA Concentration: 9.333 pg/µl
rRNA Ratio [28s / 18s]: 0,0
RNA Integrity Number (RIN): 8 (B.02.08)
Result Flagging Color:
Result Flagging Label: RIN:8

A OCT5

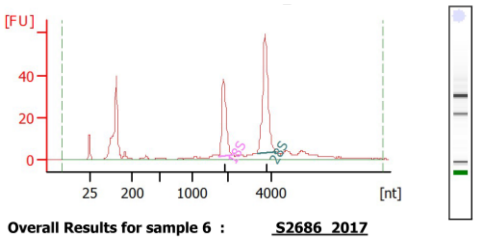

Overall Results for sample 6 : S2686_2017

RNA Area: 393,5
RNA Concentration: 187 ng/µl
rRNA Ratio [28s / 18s]: 0,0
RNA Integrity Number (RIN): 9.7 (B.02.08)
Result Flagging Color:
Result Flagging Label: RIN: 9.70

B OCT6

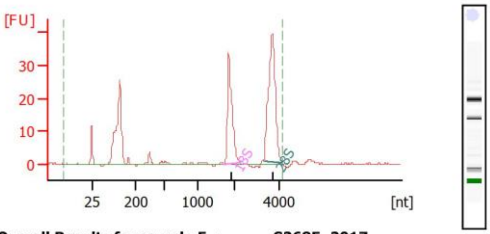

Overall Results for sample 5 : S2685_2017

RNA Area: 248,1
RNA Concentration: 118 ng/µl
rRNA Ratio [28s / 18s]: 0,0
RNA Integrity Number (RIN): 9.7 (B.02.08, Anomaly Threshold(s) manually adapted)
Result Flagging Color:
Result Flagging Label: RIN: 9.70

C OCT7

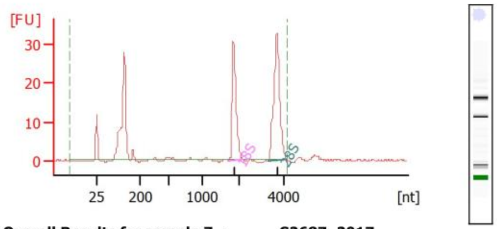

Overall Results for sample 7 : S2687_2017

RNA Area: 194,1
RNA Concentration: 92 ng/µl
rRNA Ratio [28s / 18s]: 0,0
RNA Integrity Number (RIN): 9.2 (B.02.08, Anomaly Threshold(s) manually adapted)
Result Flagging Color:
Result Flagging Label: RIN: 9.20

D OCT8

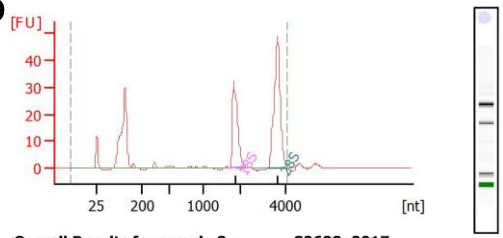

Overall Results for sample 8 : S2688_2017

RNA Area: 273,4
RNA Concentration: 130 ng/µl
rRNA Ratio [28s / 18s]: 0,0
RNA Integrity Number (RIN): 9.6 (B.02.08, Anomaly Threshold(s) manually adapted)
Result Flagging Color:
Result Flagging Label: RIN: 9.60

E OHT13

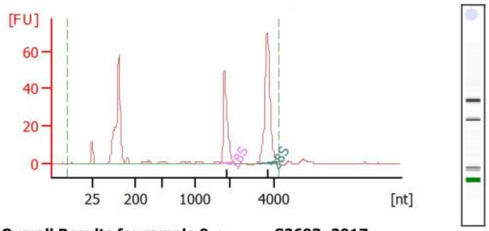

Overall Results for sample 9 : S2693_2017

RNA Area: 364,1
RNA Concentration: 173 ng/µl
rRNA Ratio [28s / 18s]: 0,0
RNA Integrity Number (RIN): 9.5 (B.02.08, Anomaly Threshold(s) manually adapted)
Result Flagging Color:
Result Flagging Label: RIN: 9.50

F OHT14

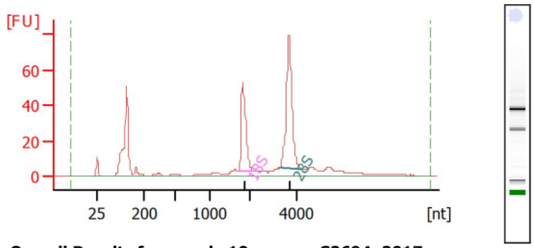

Overall Results for sample 10 : S2694_2017

RNA Area: 515,8
RNA Concentration: 245 ng/µl
rRNA Ratio [28s / 18s]: 0,0
RNA Integrity Number (RIN): 9.5 (B.02.08)

G OHT15

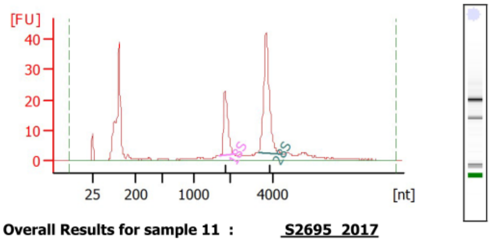

Overall Results for sample 11 : S2695_2017

RNA Area: 311,0
RNA Concentration: 148 ng/µl
rRNA Ratio [28s / 18s]: 0,0
RNA Integrity Number (RIN): 9.4 (B.02.08)
Result Flagging Color:
Result Flagging Label: RIN: 9.40

H OHT16

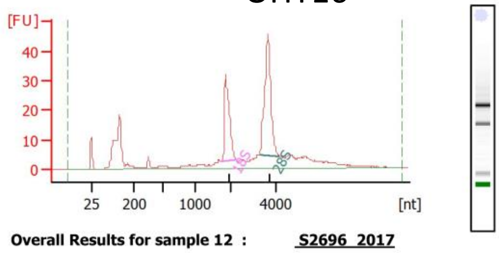

Overall Results for sample 12 : S2696_2017

RNA Area: 368,6
RNA Concentration: 175 ng/µl
rRNA Ratio [28s / 18s]: 0,0
RNA Integrity Number (RIN): 9.3 (B.02.08)
Result Flagging Color:
Result Flagging Label: RIN: 9.30
