# Supplementary material for: Deciphering sex-specific miRNAs as heat-recorders in zebrafish
Source: Sci Rep. 2022 Nov 4;12:18722. doi: 10.1038/s41598-022-21864-3 (PMC9636255; doi:10.1038/s41598-022-21864-3)
Supplement: Supplementary file 4 — Supplementary Information 4. [file 41598_2022_21864_MOESM4_ESM.pdf]

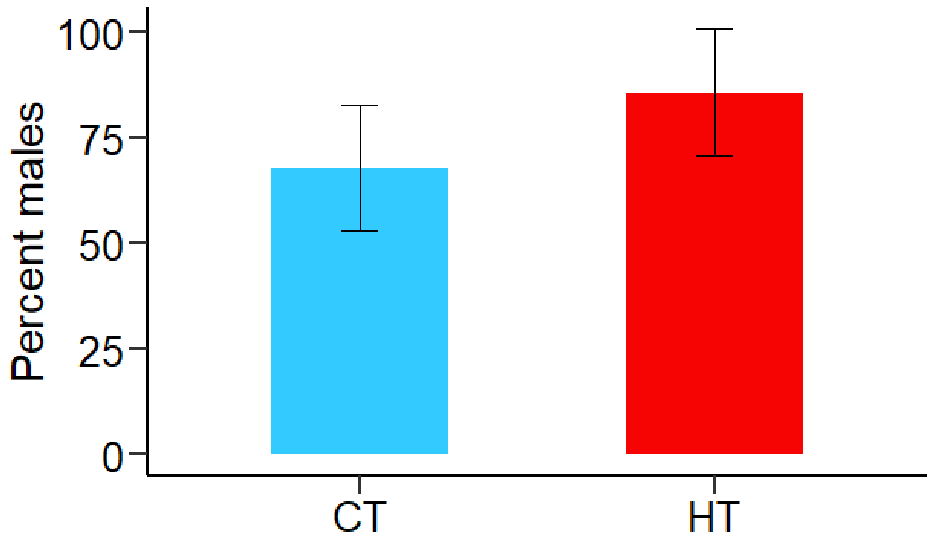

Supplemental Figure S1. Sex ratio in adult zebrafish after high temperature (34°C) treatment during sex differentiation. Results show the mean ± SD of two technical replicates of one family pair for control (CT, 28°C; n = 27) and treated (HT, 34°C; n = 52) groups. No significant differences were found between the two groups by Chi-squared test.

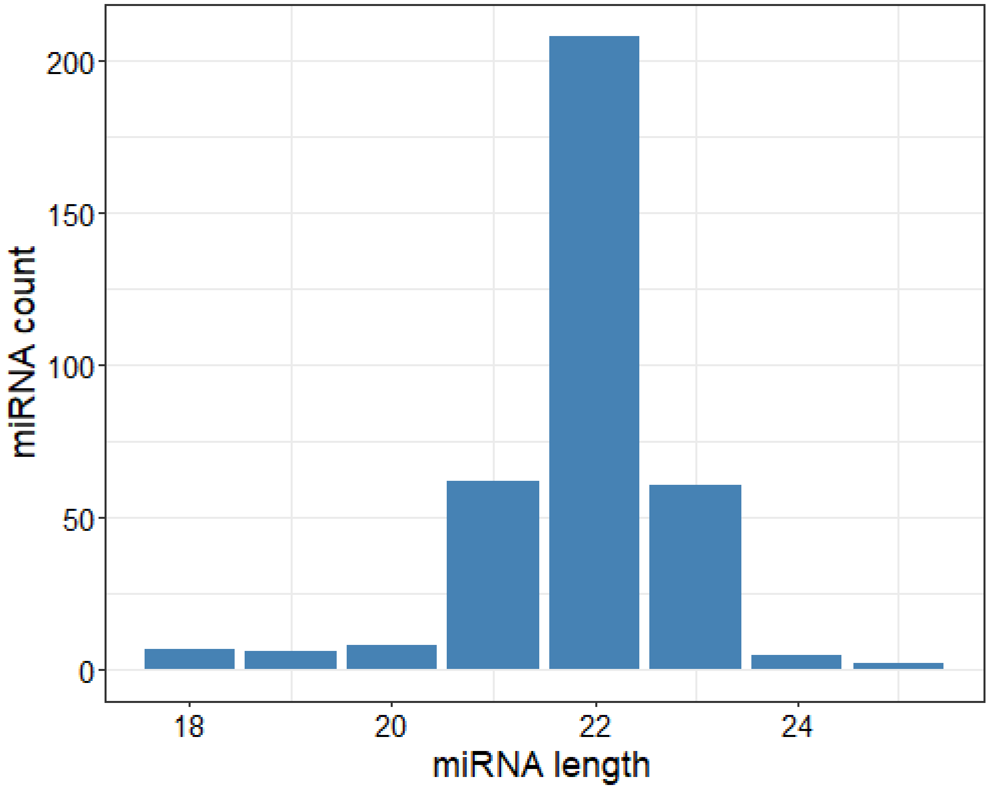

Supplemental Figure S2. Length distribution of the miRNAs obtained from the consensus sequencing alignments.

A

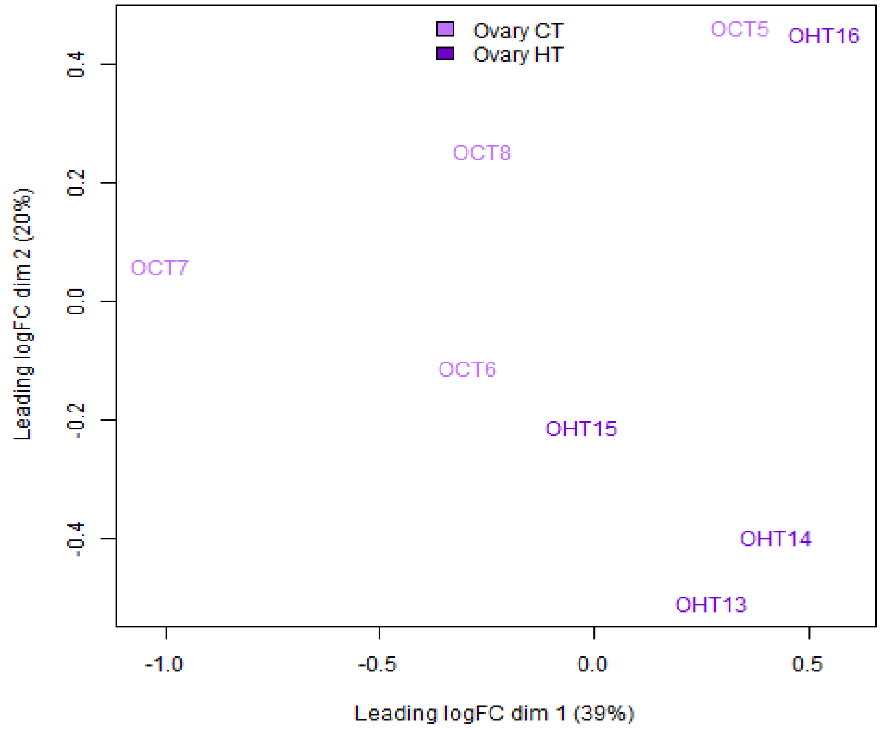

B

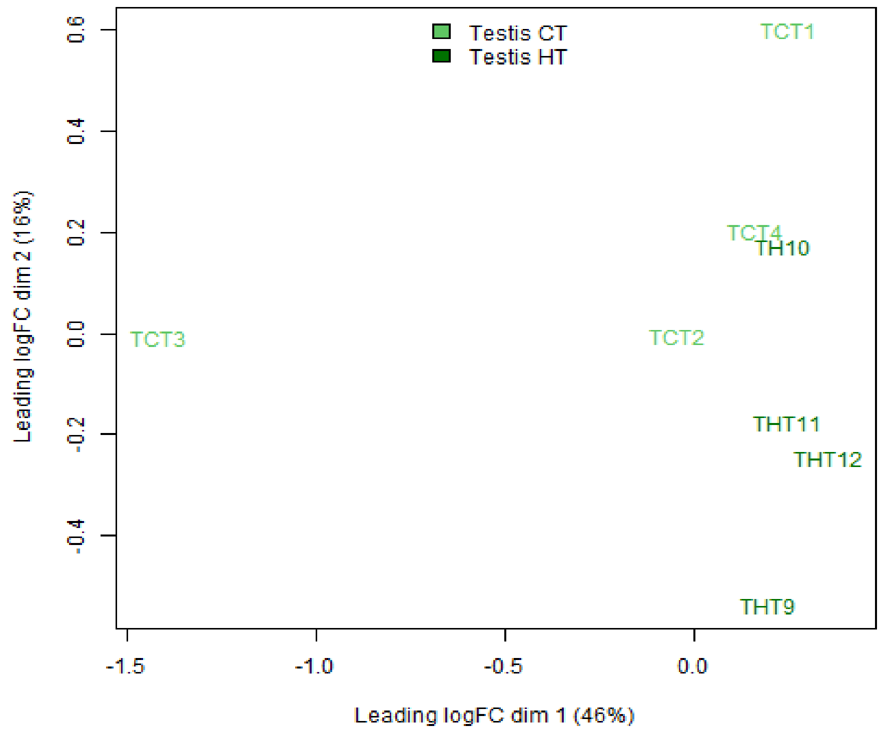

Supplemental Figure S3. Multidimensional scaling (MDS) of ovary (A) and testis (B) RNA sequencing data from 8 samples each gonadal tissue. Four in each group, ovary and testis, control and high temperature.

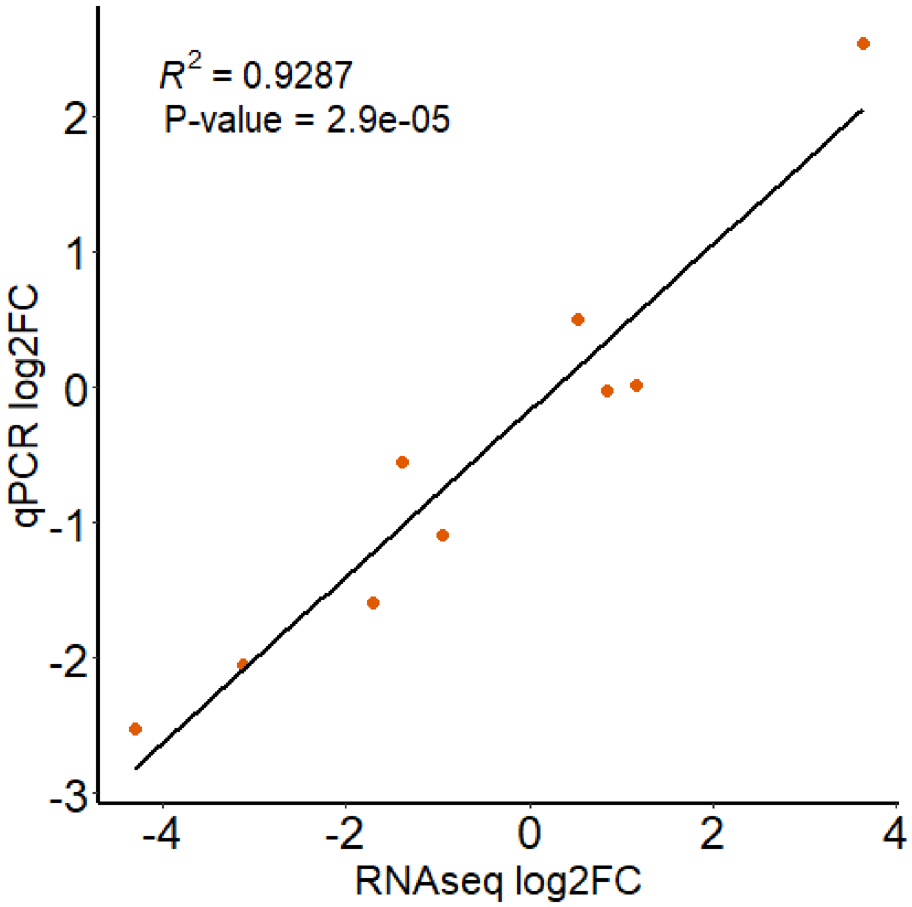

Supplemental Figure S4. Validation of RNA sequencing data by qPCR of eight miRNAs. A total of nine comparisons were used and based on the log2 fold. The miRNAs compared were dre-miR-146b-5p ovary control temperature (OCT) *vs.* testis control temperature (TCT) and ovary high temperature (OHT) *vs.* OCT, dre-miR-21-5p OHT *vs.* OCT, dre-miR-726-3p OCT *vs.* TCT, dre-miR-726-5p THT *vs.* TCT, dre-miR-92a-3p OHT *vs.* OCT, dre-miR-202-5p OHT *vs.* OCT, dre-miR-143-3p THT *vs.* TCT and dre-miR-122-5p THT *vs.* TCT.

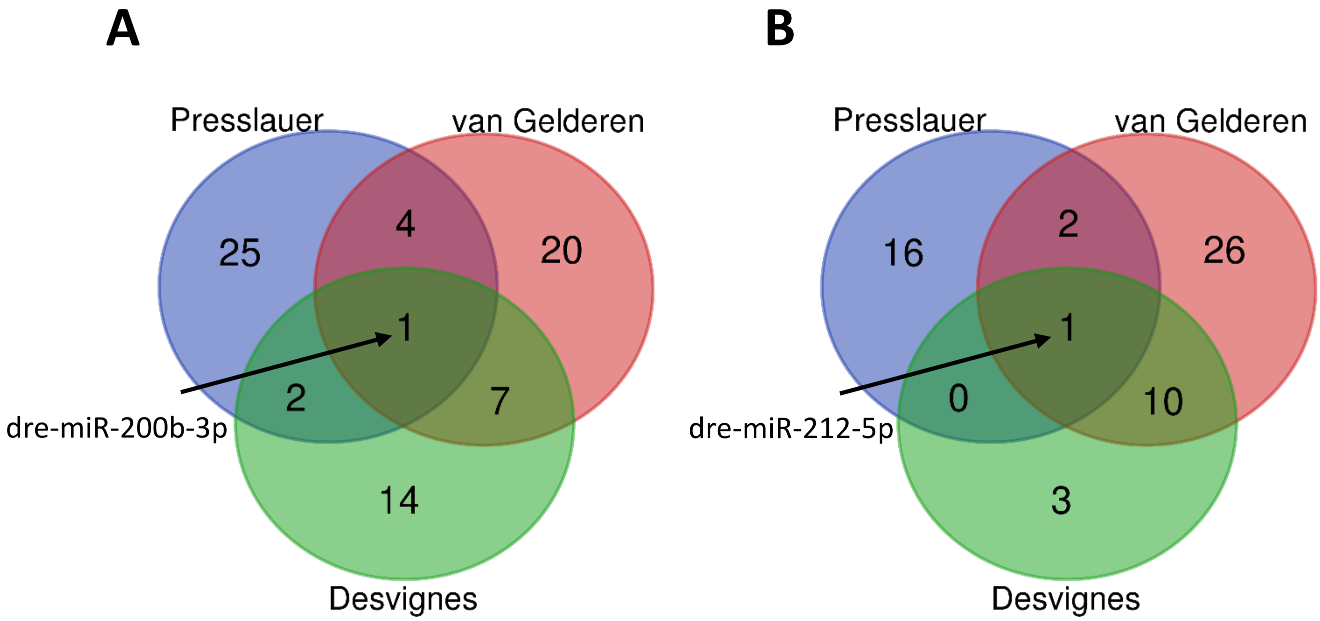

Supplemental Figure S5. Venn diagrams of differentially expressed (DE) miRNAs in ovary *vs.* testis. **A)** Common DE expressed miRNAs in ovaries between Presslauer *et al* 2019, Desvignes *et al* 2017, and present data. One miRNA was DE in all datasets: dre-miR-200b-30. **B)** Common DE expressed miRNAs in testes between Presslauer *et al* 2019, Desvignes *et al* 2017, and present data. One miRNA was DE in all datasets: dre-miR-212-5p.

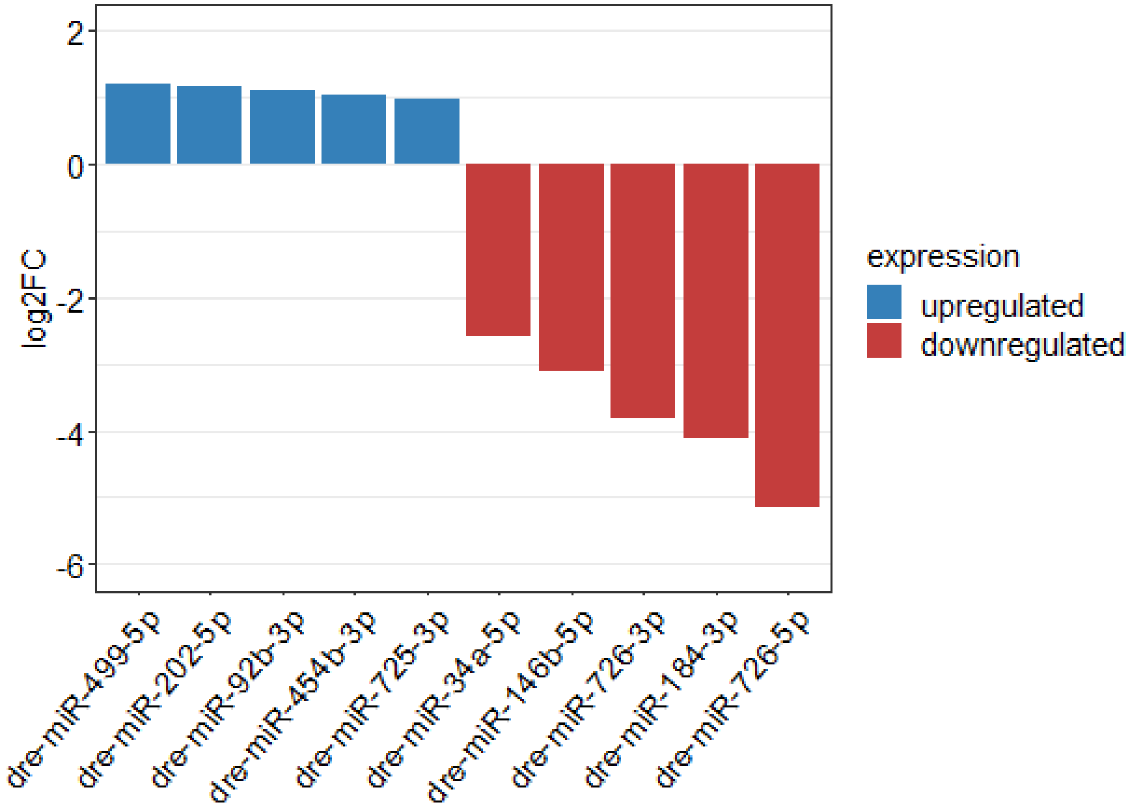

Supplemental Figure S6. Top five up- and downregulated miRNAs in adult ovaries heated with high temperature in zebrafish.

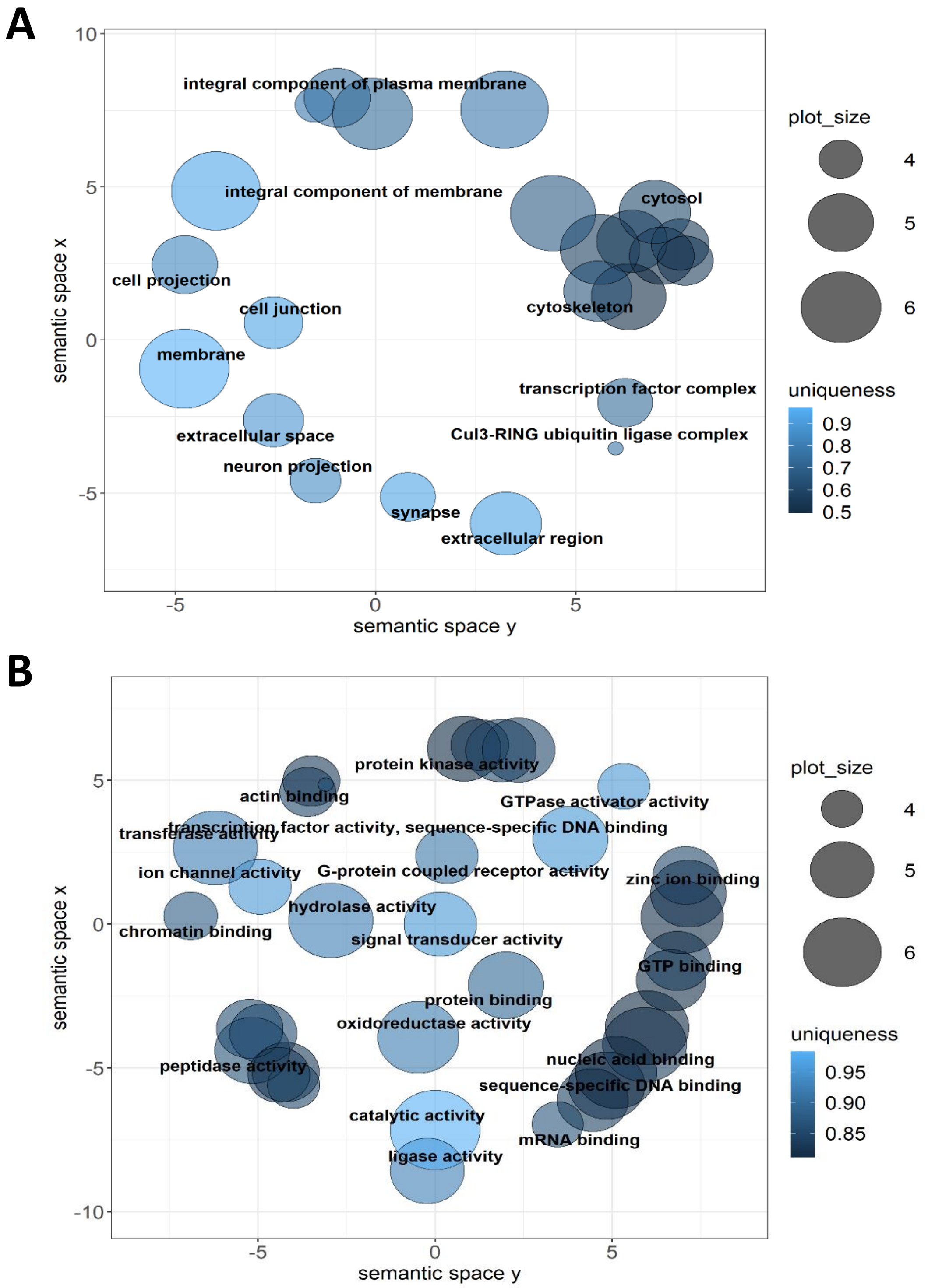

Supplemental Figure S7. Visual representation of Gene Ontology (GO) terms related to related Cellular components (CC) **A)** or to Molecular function (MF) **B)** obtained from predicted target genes of differentially expressed miRNAs in ovary. The most frequent terms were membrane, integral component of membrane and extracellular region for CC and metal ion binding, zinc ionbinding, and transferase activity for MF. Color intensity represents the frequency of the GO term as linked to the target genes. PlotSize shows the frequency of the GO term in the UniProt database. The GO terms with a dispensability of < 0.25 are annotated in the plot.
